# miR‐485's anti‐drug resistant epilepsy effects by regulating SV2A/PSD‐95 and targeting ABCC1 and neuronal signaling‐transduction proteins in hippocampus of rats

**DOI:** 10.1002/brb3.2247

**Published:** 2021-07-21

**Authors:** Kaixuan Wang, Jing Wu, Jiangping Wang, Kewen Jiang

**Affiliations:** ^1^ Department of Pediatrics Jinhua Central Hospital Jinhua China; ^2^ Department of Child Psychology National Clinical Research Center For Child Health The Children's Hospital Zhejiang University School of Medicine Hangzhou China; ^3^ Department of Rehabilitation National Clinical Research Center For Child Health The Children's Hospital Zhejiang University School of Medicine Hangzhou China

**Keywords:** drug‐resistant epilepsy, drug‐resistant‐related protein, hippocampus, miR‐485, neuronal signaling‐transduction protein, PSD‐95, SV2A

## Abstract

**Aim:**

Drug‐resistant epilepsy (DRE), most subsequently developing refractory epilepsy, causes a significant burden to the society. microRNAs have been demonstrated as key regulators and therapeutic targets in epilepsy. Accordingly, the aim of the present study was to test whether miR‐485 could be a potential target for DRE.

**Methods and results:**

An in vivo DRE model was developed in Sprague–Dawley rats by lithium chloride‐pilocarpine and screened by antiepileptic drugs. We found that miR‐485‐5p in hippocampus was significant downregulated at early stage and recovered to normal level at late stage of DRE. Overexpression of miR‐485‐5p in dentate gyrus (DG) of hippocampus in DRE rats could significantly decrease the frequency of seizures and the numbers of epileptiform spikes of hippocampal DG neuron, and could specifically decrease SV2A expression without affecting PSD‐95 expression in DG. Furthermore, miR‐485‐5p overexpression could significantly downregulate the expression of efflux transporter related to multidrug resistance (ABCC1) in hippocampus at late stage of DRE. Finally, a specific expression pattern of neuronal signaling‐transduction proteins (LRP4, MDM4, p53, and TMBIM1) for DRE was observed, and miR‐485‐5p overexpression could modulate these proteins’ expression levels toward normal in hippocampus both at early and late stage of DRE.

**Conclusion:**

Collectively, these results suggest that miR‐485 was a potential target for anti‐DRE, and this effects might be partially via miR‐485‐5p/homeostatic‐synaptic plasticity‐molecule axis and/or targeting efflux transporter (ABCC1) and other neuronal signaling‐transduction proteins (LRP4, MDM4, p53, and TMBIM1).

## Graphical abstract

MiR‐485 was a potential targets for anti‐DRE, and this effects might be partially via miR‐485‐5p/homeostatic‐synaptic plasticity‐molecule axis and/or targeting efflux transporter (ABCC1) and other neuronal signaling‐transduction proteins (LRP4, MDM4,p53 and TMBIM1).

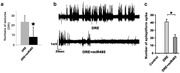

## 1 INTRODUCTION

Epilepsy, a chronic noncommunicable disease of the central nervous system (CNS) that affects 7% people of all ages. Nearly 30–40% of the patients are drug‐resistant epilepsy (DRE), being pharmacoresistant to two or more types of antiepileptic drugs (AEDs) and causing a significant burden to patients, their families, and the society (Tang et al., 2017). Although lots of new AEDs have been developed in the past few decades, the therapeutic efficacy is still limited (Tang et al., 2017). Most DRE patients subsequently develop refractory epilepsy (Kwan & Brodie, 2000; Kwan et al., 2010) and suffer from severe psychosocial problems and even increased morbidity and mortality (French, 2007). It has been proposed that refractory epilepsy could be mediated by several mechanisms relating to seizure severity, pharmacokinetic, gene variant, neural network, and the transporter such as the efflux transporter correlates with pharmacoresistance (Potschka, 2012; Tang et al., 2017).

Recently, microRNAs (miRNAs), a class of endogenous short noncoding RNAs that modulate gene expression at the posttranscriptional level, inducing the degradation or inhibition of the translation of target genes (Bartel, 2009; Gisel et al., 2014; Shukla et al., 2011), have received increasing attention. Results of previous studies suggested a possible role of miRNAs in epilepsy's pathophysiology since epileptogenic and molecular profiles found a number of miRNAs changed in epileptic hippocampus of both animal models and human tissues, and it was also proposed as therapeutic targets for epilepsy. Furthermore, recent study results have indicated a role of miRNAs in reversing pharmacoresistance in the CNS of epilepsy (Liu et al., 2012; Xie et al., 2018). miR‐485 is one of miRNAs that significantly downregulated in hippocampus of epilepsy (Gorter et al., 2014), and miR‐485 is critical in controlling homeostatic synaptic plasticity (Cohen et al., 2011). Homeostatic synaptic plasticity is also one of the key epileptogenesis mechanisms, and we tested the anti‐DRE effects of miR‐485 and whether these effects were via regulating homeostatic synaptic plasticity molecules and/or targeting efflux transporter and other neuronal signaling‐transduction proteins.

## 2 MATERIALS AND METHODS

### 2.1 Ethical statement

The Sprague–Dawley (SD) rats were bought from the Zhejiang University Animal Center. All procedures were approved by the Zhejiang University Animal Experiment Ethics Committee and followed the Zhejiang University Guidelines on Animal Care.

### 2.2 DRE model

The DRE model was induced by lithium chloride‐pilocarpine and screened by ADEs. Briefly, after weighing (100–150 g), rats were injected intraperitoneally (i.p.) with lithium chloride (127 mg/kg), and at 18.5–23.5 h, they were treated with methylscopolamine bromide (1 mg/kg, i.p.) then followed with pilocarpine (60 mg/kg) 30 min later. The behavior after pilocarpine injection was observed and the seizure degree of each rat was assessed according to the Racine criteria. The status epilepticus (SE) was defined as a continuous generalized seizure (grade ≥ 4 seizures). The onset latency, that is, from the time when the drug was administered to the onset of SE, was recorded. It should repeat pilocarpine administration if there was no SE attack 30 min after pilocarpine injection. Ninety minutes after SE, diazepam was given (i.p.) to stop seizures. Rats with seizures of grade < 4 were excluded. No spontaneous seizures were excluded in 2 weeks. Two weeks after SE, phenytoin sodium (PHT, 50 mg/kg·d) was injected via the tail vein, and observed for 1 week after the PHT injection. If the rats still showed seizures and were treated with PHT (70 mg /kg·d) again, and observed for another week, and those rats still showed seizures, they were deemed as the successful model of DRE. In the lithium chloride‐pilocarpine (i.e., epilepsy) group, PHT was replaced by equal volume saline; in the control group, rats were only injected with equal volume saline at the same time points as those in the DRE group.

### 2.3 Real‐time RT‐PCR

Total RNA was extracted with TRIzol reagent (Invitrogen, Shanghai, China), and cDNA was synthesized using RevertAid First Strand cDNA synthesis Kit (Thermo). Quantitative real‐time RT‐PCR analysis was performed by StepOne Plus (Applied Biosystems) and GoTaq qPCR Master Mix (Promega). The primers used for miR‐485 were obtained from Invitrogen (Shanghai, Rat miR‐485‐5p forward: 5′‐ ACACTCCAGCTGGGAGAGGCTGGCCGTGAT‐3′; Rat miR‐485‐5p reverse: 5′‐ CTCAACTGGTGTCGTGGAGTCGGCAATTCAGTTGAGGAATTCAT‐3′). Data were normalized by the rat U6 expression in each sample.

### 2.4 Western blots

Rat hippocampus was lysated in RIPA Lysis Buffer (Beyotime) containing (Biyuantian, Wuhan, China). Protein concentrations of each sample were measured by BCA assay. Equal protein amounts were subjected to SDS‐PAGE and transferred onto PVDF membranes. After incubation with the primary antibodies—anti‐GAPDH (1:10000, 2118S; Cell Signaling Technology), anti‐LRP4 (1:2000, DF9610; Affinity), anti‐ABCC1 (1:1000, DF7148; Affinity), anti‐MDM4 (1:1000, DF7532; Affinity), anti‐p53 (1:2000, AF0879; Affinity), anti‐TMBIM1 (1:1000, DF4580; Affinity), anti‐PSD‐95 (6G6‐1C9, 1:250; Abcam), and anti‐SV2A (1:100; DSHB) overnight at 4°C—the membranes were incubated with second antibodies (goat anti‐rabbit IgG, HRP‐conjugate, 1:5000, BL003A; Biosharp) at room temperature for 2 h. Chemiluminescent values of the protein of interest were divided by its corresponding GAPDH chemiluminescent values.

### 2.5 Electrophysiology

Hippocampal slices (350 μm) from rats were prepared in ice‐cold solution containing (in mM) 234 Sucrose, 2.5 KCl, 0.5 CaCl_2_, 10 MgCl_2_, 1.25 NaH_2_PO_4_·2H_2_O, 26 NaHCO_3_, and 11 D‐Glucose equilibrated with 95% O_2_–5% CO_2_, with a vibratome (Leica, VT1000s). Slices were continuously incubated in standard artificial cerebrospinal fluid (ACSF; 30 min, 34°C) equilibrated with 95% O_2_–5% CO_2_. Before recording, the slices were maintained at room temperature for at least 40 min. Slices were transferred to a submerged recording chamber perfused with ACSF equilibrated with 95% O_2_–5% CO_2_ at a rate of 2∼3 ml/min.

Excitatory postsynaptic potentials (EPSPs) and epileptiform spikes were recorded with glass electrodes (3∼5 MΩ tip resistance) filled with 120 mM cesium methanesulfonate, 2 mM MgCl_2_, 5 mM EGTA, 10 mM Hepes, 5 mM ATP, and 0.5 mM GTP, at 290 mOsm (adjusted with cesium hydroxide), pH7.4. Recordings were filtered at 2 kHz, sampled at 20 kHz with a multiclamp 700B amplifier (Molecular Devices), and acquired with a Digidata‐1440A digitizer and pClamp 10.2 software (Molecular Devices). Whole‐cell current clamp recording for each hippocampal DG neuron lasted for 1 h. EPSPs and epileptiform spikes were analyzed offline with pClamp10.2 software.

### 2.6 Generation of miR‐485‐5p‐overexpressing adeno‐associated virus and stereotaxic injection

The miR‐485‐5p‐overexpressing adeno‐associated virus (AAV) (AAV‐miR‐485‐5p) was provided by Hanbio Biotechnology Co., Ltd. (China). Briefly, the AAV transgene plasmid pAAV‐CMV‐miR‐485‐5p‐ires‐hrEGFP was constructed by cloning pre‐miR‐485‐5p into the AAV vector pAAV‐CMV‐ires‐hrEGFP with *Bam*HI and *Eco*RI restriction sites. At 72 h after the co‐transfection of pAAV‐RC9, pHelper, and the AAV transgene plasmid, the HEK293 cells were incubated with Benzonase endonuclease (Sigma, USA) at 37°C for 1 h. The AAV particles were purified by a heparin column (Sigma), concentrated using Amicon Ultra‐4 (Millipore, USA), and aliquoted and stored at −80°C.

DRE rats (5 weeks after SE) were anesthetized (sodium pentobarbital, 60 mg/kg, i.p.) and received stereotaxic injections into the hippocampal DG (+3 mm AP, +2 mm ML, and −3.5 mm DV) using a frame (Kopf Instruments, USA). AAV‐miR‐485‐5p (2 μl) or AAV‐GFP(1 μl mixed with 1 μl of 20% mannitol) was injected at a speed of 0.1 μl/min. Four weeks after the surgery, the AAV infection effects were observed by immunofluorescence microscopy. miR‐485‐5p expression in the rat hippocampal tissue was analyzed by qRT‐PCR and Western blots.

### 2.7 Histological preparation of tissue and immunostaining

Rats were terminated at 14 weeks after SE under deep isoflurane anesthesia and decapitated for rapid brain dissection on ice. After fixed in 3% paraformaldehyde overnight at 4°C and then dehydrated in cacodlyate‐PBS containing 15−30% sucrose at 4°C, brains were processed for cryo‐sectioning at 40 μm thickness (coronal/sagittal) on microtome. Free‐floating brain sections were mounted on glass slides and imaged for AAV‐GFP or stored in glycerol at −20°C for immunofluorescent staining. After antigen‐retrieval by incubating sections in 0.01 M sodium citrate buffer (pH 8.45) for 20 min, sections were blocked in donkey serum (Miller et al., 2011). Primary antibodies diluted in 0.1% triton‐X containing tris‐base‐saline (TBS) are NeuN (1:1000, MAB377; Merck), PSD‐95 (6G6‐1C9, 1:500; Abcam), and SV2A (1:300; DSHB). Sections were stained for DAPI (Sigma) and mounted onto glass coverslips in VectaShield mounting medium and stored at 4°C until imaging.

For immunohistochemistry, free‐floating sections were rinsed three times for 15 min each in buffer (TBS, pH6.8−7.4), followed by quenching of endogenous peroxidases in 3% hydrogen peroxide, treatment in blocking serum (3%), incubation in primary antibody (SV2A, 1:150; PSD‐95,1:500) overnight at 4°C, and incubation in secondary antibody for 2 h at room temperatures. Buffer washes (TBS) were interposed between all basic steps. Biotinylated secondary antibody, avidin–biotin amplification, and chromogen development system was used for chromogen‐labeled tissue (VECTASTAIN ABC Elite kit; Vector Laboratories). Once the reaction was complete, sections were mounted onto gelatin‐subbed slides, dried overnight, dehydrated in graded alcohols, cleared in xylene, and coverslipped.

### 2.8 Statistical analyses

All results are expressed as the mean ± SD. Statistical results were analyzed using SPSS 19.0 software (SPSS, USA). Differences between groups in the levels of Western blot and qPCR were analyzed using unpaired Student's *t*‐test. A value of *p* < .05 was considered statistically significant.

## 3 RESULTS

### 3.1 The dynamic expressions of miR‐485 in hippocampus of DRE rats

**FIGURE 1:**
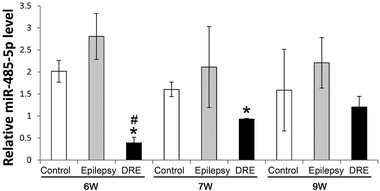
The dynamic expression of miR‐485 mRNA in hippocampus of DRE rats. *p < .05 versus the control group; ^#^ *p *< .05 versus the epilepsy group

miR‐485 is one of the significantly downregulated miRNAs in hippocampus of epilepsy and is critical in controlling homeostatic synaptic plasticity in the brain. We asked whether the miR‐485‐5p was changed in hippocampus of DRE rats. We found that the mRNA level was significantly downregultaed in DRE rats at 6 weeks (*n* = 6) and 7 weeks (*n* = 3) after SE compared to the control (6 weeks and 7 weeks, *n* = 3) and epilepsy (6 weeks and 7 weeks, *n* = 3) groups, respectively (*p *< .05, Figure 1), but the level recovered to the normal level at 9 weeks (*n* = 3) after SE, which was not significantly different from that of the control (*n* = 3) and epilepsy (*n* = 3) groups, respectively (*p *> .05, Figure 1).

### 3.2 miR‐485‐5p overexpression in hippocampus decreased epileptic‐discharges of DRE rats

**FIGURE 2:**
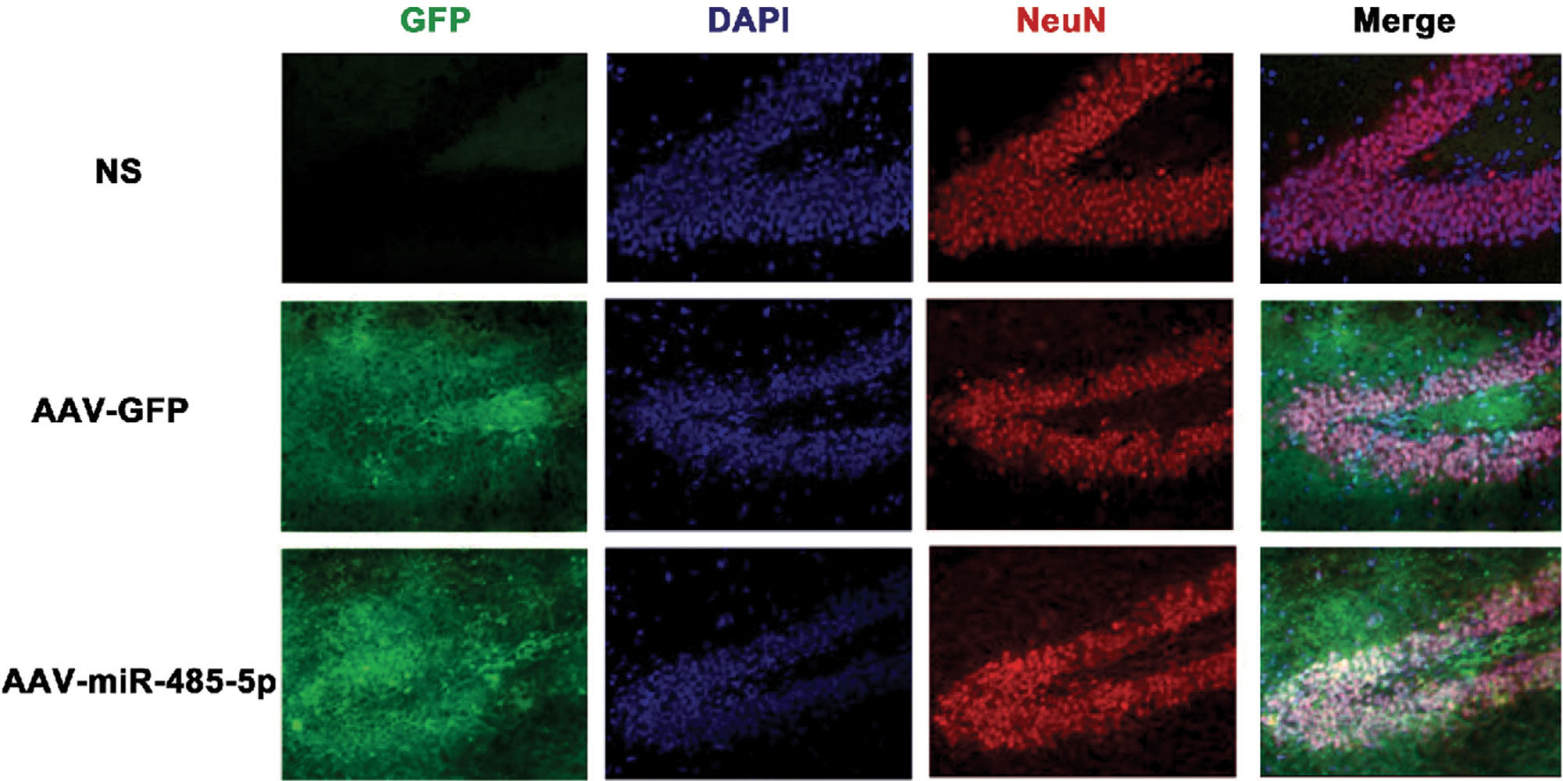
The AAV effectively infected the hippocampus as indicated by immunofluorescence microscopy (200×)

**FIGURE 3:**
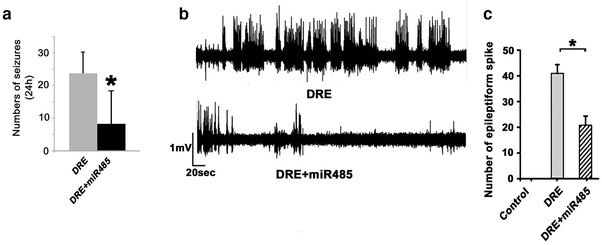
miR‐485‐5p overexpression decreased the epileptic‐discharges of DRE rats. (a) The numbers of seizures (grade ≥ 4) in 12 h/d*7 were significantly decreased by the AAV‐miR‐485‐5p infection at 14 weeks after SE (*n* = 4, *p *< .05). (b and c) miR‐485‐5p overexpression decreased the epileptic‐discharges of hippocampal neurons of rats with DRE. (b) Samples of whole cell current recording using hippocampal slices of DRE rats. (c) Barographs of the numbers of epileptiform spikes in hippocampal slices of DRE rats (15 neurons were recorded from three rats for each group). * *p *< .05

To further determine whether miR‐485‐5p could inhibit the epileptic‐discharges of DRE rats, AAV‐miR‐485‐5p was injected into DG of DRE rats using a stereotaxic frame. Four weeks after injection, the AAV effectively infected the brain tissue as indicated by immunofluorescence microscopy (Figure 2). Saline and AAV‐GFP served as controls. At 14 weeks after SE, AAV‐miR‐485‐5p‐infected DRE rats were subjected to seizure evaluation using video‐recording for 12 h/d*7 and whole‐cell current recording using hippocampal slices. We found that the numbers of seizures ( grade ≥ 4) were significantly decreased by the AAV‐miR‐485‐5p infection (*n* = 4, *p *< .05, Figure 3a). The numbers of epileptiform spikes were also significantly reduced by the AAV‐miR‐485‐5p infection of DG neurons (*p *< .01, 15 neurons were recorded from three rats for each group) (Figure 3b and c).

### 3.3 miR‐485‐5p overexpression affected expressions of homeostatic synaptic plasticity‐related proteins in hippocampus of DRE rats

**FIGURE 4:**
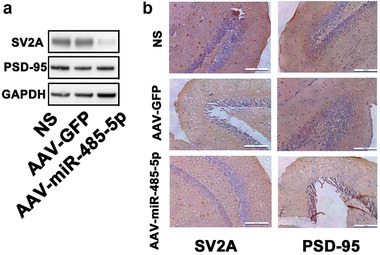
The effects of miR‐485‐5p overexpression on expressions of homeostatic‐synaptic‐plasticity‐related proteins in hippocampus of DRE rats. (a) Samples of Western blotting bands. (b) Immunohistochemistry stainings of DG in hippocampus of DRE rats (14 weeks after SE, 200×)

miR‐485 is critical in controlling homeostatic synaptic plasticity (Cohen et al., 2011) via targeting SV2A to regulate dendritic spine density and PSD‐95 clustering. Homeostatic synaptic plasticity is also one of the key epileptogenesis mechanisms, and we determined the effects of miR‐485‐5p overexpression on expressions of PSD95 and SV2A in hippocampus of DRE rats. AAV‐miR‐485‐5p infection specifically decreased SV2A expressions without affecting PSD‐95 expressions in DG of DRE rats (14 weeks after SE, Figure 4a). Immunohistochemistry staining further demonstrated that AAV‐miR‐485‐5p infection decreased SV2A expressions without affecting PSD‐95 expressions in DG of DRE rats (14 weeks after SE, Figure 4b, 200×).

### 3.4 miR‐485‐5p overexpression affected expressions of multidrug resistance‐associated protein (MRP1/ABCC1) and neuronal signaling‐transduction proteins in hippocampus of DRE rats

**FIGURE 5:**
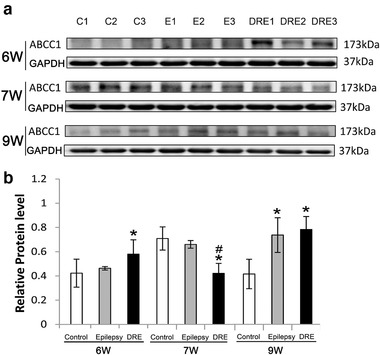
The expressions of drug‐resistant‐related proteins (ABCC1) in hippocampus of DRE rats at 6, 7, and 9 weeks after SE. *p < .05 versus the control group; ^#^ *p *< .5 versus the epilepsy group

Multidrug resistance due to efflux transporters has been studied extensively. ABCC1 is one of the best understood efflux transporters. Compared with the control group, at 6 weeks after SE, the expression levels of ABCC1 in epilepsy group were not significantly increased (*p* > .05, Figure 5a and b), but significantly increased in the DRE group (*p* < .05 Figure 5a and b); at 7 weeks after SE, the levels in epilepsy group were similar to that of the control group (*p* > .05 Figure 5a and b), but significantly decreased in the DRE group (*p* < .05 Figure 5a and b); at 9 weeks after SE, the levels in both epilepsy and DRE groups were significantly increased (*p *< .05 Figure 5a and b).

**FIGURE 6:**
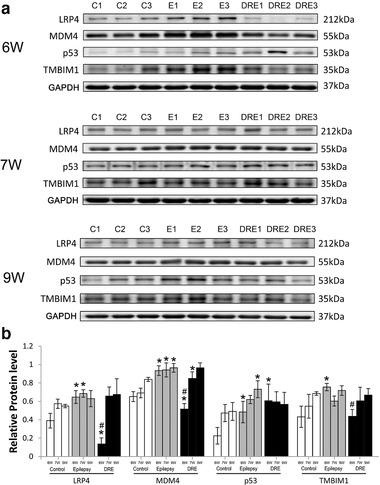
The expression of neuronal signaling‐transduction proteins in hippocampus of DRE rats at 9 weeks after SE. (a) Western blotting bands; (b) barographs of expression levels. *p < .05 versus the control group; ^#^ *p *< .05 versus the epilepsy group

We reviewed literatures and selected four cell signaling‐transduction proteins (LRP4, MDM4, p53, and TMBIM1) to check their changes in hippocampus of DRE rats. At 6 weeks after SE, compared with the control group, the expression levels of MDM4, p53, and TMBIM1 were significantly increased in the epilepsy group (*p* < .05, Figure 6a and b), while the levels of LRP4 and MDM4 were significantly decreased (*p* < .05, Figure 6a and b) but the levels of p53 were significantly increased in the DRE group (*p* < .05, Figure 6a and b), and the levels of LRP4, MDM4 and TMBIM1 were significantly downregulated in the DRE group compared with the epilepsy group (*p* < .01, Figure 6 a and b). At 7 weeks after SE, compared with control group, the levels of LRP4 and MDM4 were significantly increased in epilepsy group (*p* < .05, Figure 6a and b), while only MDM4 was significantly increased in the DRE group (*p* < .05, Figure 6a and b), and the levels of LRP4, MDM4, p53, and TMBIM1 had no significant changes in the DRE group compared with the epilepsy group (*p* > .05, Figure 6a and b). At 9 weeks after SE, compared with the control group, the levels of MDM4 and p53 were significantly increased in the epilepsy group (*p* < .05, Figure 6a and b); the levels of MDM4 were significantly increased in the DRE group (*p* < .05, Figure 6a and b), and the levels of LRP4, MDM4, p53, and TMBIM1 in the DRE group had no significant difference compared with the epilepsy group (*p* > .05, Figure 6a and b).

**FIGURE 7:**
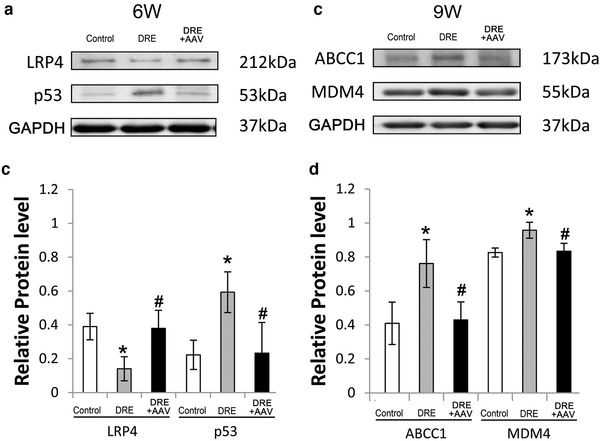
miR‐485‐5p overexpression modulated the expression of drug‐resistant‐related proteins (ABCC1) and neuronal signaling‐transduction proteins (LRP4, p53, and MDM4) in hippocampus of DRE rats. (a and c) Western blotting bands; (b and d) barographs of expression levels. *p < .05 versus the control group; ^#^ *p *< .05 versus the epilepsy group

We then tested whether miR‐485‐5p overexpression affected expressions of ABCC1 at 9 weeks after SE and expressions of cell signaling‐transduction proteins (LRP4 and p53 at 6 weeks after SE, and MDM4 at 9 weeks after SE) in hippocampus of DRE rats. We found miR‐485‐5p overexpression could significantly modulate the levels of ABCC1, LRP4, MDM4, and p53 toward normal in hippocampus of DRE rats (*p* < .05 Figure 7a–d).

## 4 DISCUSSION

Most DRE patients subsequently develop refractory epilepsy (Kwan & Brodie, 2000; Kwan et al., 2010) and suffer from severe psychosocial problems (French, 2007) due to therapy being still limited. It would be of a great significance to find a novel target for DRE and its relevant mechanisms. Recently, miRNAs that modulate gene expression at the posttranscriptional level, inducing the degradation or inhibition of the translation of target genes (Bartel, 2009; Gisel et al., 2014; Shukla et al., 2011), have received increasing attention. Increasing evidences have suggested that miRNAs played an important role in epilepsy's pathophysiology and might be potential therapeutic targets for epilepsy. Furthermore, studies have demonstrated that miRNAs could directly reverse pharmacoresistance in the epileptic CNS (Liu et al., 2012; Xie et al., 2018). miR‐485 is one of miRNAs that significantly downregulated in epileptic hippocampus (Gorter et al., 2014), and is critical in controlling homeostatic synaptic plasticity (Cohen et al., 2011). Homeostatic synaptic plasticity is also one of the key mechanisms of epileptogenesis; herein we checked the miR‐485′s anti‐DRE effects and whether these effects were via regulating homeostatic synaptic plasticity.

In the present study, we found that miR‐485‐5p in hippocampus of DRE was significantly downregulated at early stage and recovered toward normal at late stage, while its levels were not significantly changed in the epilepsy group. Our results showed that in the epilepsy group, miR‐485‐5p was not significantly changed in hippocampus. This result is different from the previous reports (Gorter et al., 2014), which might be due to different methods of model preparation, observation time points, etc. Then we tested whether miR‐485‐5p overexpression at early stage could inhibit the genesis of DRE? We found that miR‐485‐5p overexpression in DG of DRE rats did reduce the numbers of epileptiform spikes (*p* < .01) (Figure 7a and b). Our results indicated that miR‐485‐5p was a potential targets for anti‐DRE.

Previous study has demonstrated that miR‐485 was critical in controlling homeostatic synaptic plasticity (Cohen et al., 2011) via targeting SV2A to regulate dendritic spine density and PSD‐95 clustering. Homeostatic synaptic plasticity is also one of the key epileptogenesis mechanisms; we then determined the effects of miR‐485‐5p overexpression on the expressions of PSD95 and SV2A in hippocampus of DRE rats. We found that miR‐485‐5p overexpression (AAV‐miR‐485‐5p infection) specifically decreased SV2A expression without affecting PSD‐95 expression in DG of DRE rats (14 weeks after SE). Immunohistochemistry staining further demonstrated that miR‐485‐5p overexpression decreased SV2A expression without affecting PSD‐95 expression in DG of DRE rats (14 weeks after SE). Further experiments with electrophysiology need to clarify the effects of miR‐485‐5p on homeostatic synaptic plasticity of DRE and its underlying mechanisms. In addition, PSD‐95, as a hallmark of excitatory synapses, indicates the synaptic maturation. In our experiments, miR‐485‐5p overexpression did not affect the PSD‐95 expression. Present results indicated that miR‐485 might target SV2A to control homeostatic synaptic plasticity (Cohen et al., 2011). It has been demonstrated that SV2A knockdown could mimic the effects of miR‐485 overexpression, and inhibiting endogenous miR‐485 could block the homeostatic reduction in spine density, PSD‐95 puncta density, and GluR2 expression (Cohen et al., 2011). As these morphological changes were significantly reduced after increasing synaptic activity, it was supposed that the effects of miR‐485 on synapse‐associated transcripts, including SV2A, are consistent with a possible role of this miRNA in homeostatic synaptic plasticity acting to reduce transcripts and/or suppress translation to decrease synaptic connectivity under conditions of persistent hyperactivity such as DRE.

The transporter mechanism is the most widely accepted and investigated theory for refractory epilepsy. It is postulated that transporters actively transport substrates, including AEDs, against their concentration gradient out of neurons and blood–brain barrier, limiting their entry into the neurons or brain, and thereby causing resistance (Sisodiya et al., 2006). Previous study found that transporter mRNA was overexpressed in brain tissue resected from DRE patients. Therefore, this hypothesis is based on that overexpression of efflux transporter correlates with pharmacoresistance in DRE, and ASDs are subject to active transport by efflux transporters (Sisodiya et al., 2006). ABCC1 is one of the best understood efflux transporters. In the present study, we found that ABCC1 was significantly downregulated at middle stage and markedly upregulated at late stage in hippocampus of DRE rats. miR‐485‐5p overexpression could significantly downregulate the expression of ABCC1 at late stage in hippocampus of DRE rats. Recent report provided evidence emphasizing the inhibitory effect of miR‐139‐5p on resistance to AEDs by targeting MRP1 (Wang et al., 2020). Our results also indicated that the anti‐DRE effects of miR‐485‐5p overexpression in DG neurons might partially be via targeting efflux transporters (such as ABCC1).

We reviewed literatures and selected four cell signaling‐transduction proteins (LRP4, MDM4, p53, and TMBIM1) to test their changes in hippocampus of DRE rats. We did found a specific expression pattern for DRE, as LRP4 significantly downregulated at early stage and recovered toward normal at late stage, MDM4 significantly downregulated at early stage and significantly upregulated at late stage, p53 significantly upregulated at early stage and down toward normal at late stage, and TMBIM1 did not significantly change.

LRP4 plays crucial roles in the adult CNS, including formation of neuromuscular junctions in the peripheral nervous system, synaptogenesis in the developing brain, maintenance of excitatory synaptic transmission, hippocampal synaptic plasticity, fear conditioning, associative and spatial learning, and long‐term potentiation (Gomez et al., 2014; Pohlkamp et al., 2015; Sun et al., 2016; Karakatsani et al., 2017). Moreover, LRP4 protein has been detected in postsynaptic membrane fractions purified from adult rat forebrain where it interacts with the postsynaptic scaffold protein PSD95 (Gomez et al., 2014; Tian et al., 2006). Here, we found miR‐485‐5p overexpression could significantly up‐regulate the expression of LRP4 at early stage in hippocampus of DRE rats. Our results indicated the anti‐DRE effects of miR‐485‐5p overexpression in DG neurons might partially be via targeting LRP4 to modulate the synaptogenesis, excitatory synaptic transmission, synaptic plasticity, and postsynaptic PSD95 cluster in DRE hippocampus, which need further experiments to clarify.

P53 is a well‐known tumor suppressor that has emerged as an important player in neuronal signaling‐transduction. Previous study showed that miR‐125b mimic partially protected neurons against neuroinflammation and aberrant p53 network activation‐induced apoptosis during ischemia reperfusion injury (Xie et al., 2018). Here, we showed that miR‐485‐5p overexpression could be used against DRE and significantly downregulate the expression of p53 at early stage in hippocampus of DRE rats. Our results indicated the anti‐DRE effects of miR‐485‐5p overexpression in DG neurons might partially be via targeting p53 to modulate the neuronal signaling‐transduction in DRE.

In summary, we induced the DRE model in rats by lithium chloride‐pilocarpine and screened by ADEs, and found that miR‐485‐5p in hippocampus was significant downregulated at early stage of DRE and recovered toward normal at late stage. Overexpression of miR‐485‐5p in DG of hippocampus could significantly inhibit the frequency of seizures and decrease the numbers of epileptiform spikes of hippocampal DG neuron. miR‐485‐5p overexpression specifically decreased SV2A expression without affecting PSD‐95 expression in DG of DRE rats. Furthermore, miR‐485‐5p overexpression could significantly downregulate the expression of efflux transporter related to multidrug resistance (ABCC1) at late stage in hippocampus of DRE rats. Finally, we did found a specific expression pattern of neuronal signaling‐transduction proteins (LRP4, MDM4, p53, and TMBIM1) for DRE, and miR‐485‐5p overexpression could modulate these proteins’ expression toward normal both at early or late stage in hippocampus of DRE rats. Our results indicated that miR‐485 was one of the potential targets for anti‐DRE, and its anti‐DRE effects might be partially via controlling homeostatic synaptic plasticity and/or via targeting efflux transporters (ABCC1) and other neuronal signaling‐transduction proteins (LRP4, MDM4, and p53).

## CONFLICT OF INTEREST

The author declare that there is no conflict of interest.

### PEER REVIEW

The peer review history for this article is available at https://publons.com/publon/10.1002/brb3.2247.
